# Discovering unique tobacco use patterns among Alaska Native people

**DOI:** 10.3402/ijch.v72i0.21208

**Published:** 2013-08-05

**Authors:** Julia A. Dilley, Erin Peterson, Vanessa Y. Hiratsuka, Kristen Rohde

**Affiliations:** 1 Program Design and Evaluation Services, Multnomah County Health Department and Oregon Health Authority, Portland, OR, USA; 2 Alaska Department of Health and Social Services, Anchorage, AK, USA; 3 Southcentral Foundation, Anchorage, AK, USA

**Keywords:** Alaska/epidemiology, Smoking/epidemiology, Prevalence, Smoking/ethnology, Indians, North American, Tobacco, smokeless

## Abstract

**Background:**

Alaska Native people are disproportionately impacted by tobacco-related diseases in comparison to non-Native Alaskans.

**Design:**

We used Alaska’s Behavioral Risk Factor Surveillance System (BRFSS) to describe tobacco use among more than 4,100 Alaska Native adults, stratified by geographic region and demographic groups.

**Results:**

Overall tobacco use was high: approximately 2 out of every 5 Alaska Native adults reported smoking cigarettes (41.2%) and 1 in 10 reported using smokeless tobacco (SLT, 12.3%). A small percentage overall (4.8%) reported using iq’mik, an SLT variant unique to Alaska Native people. When examined by geographic region, cigarette smoking was highest in remote geographic regions; SLT use was highest in the southwest region of the state. Use of iq’mik was primarily confined to a specific area of the state; further analysis showed that 1 in 3 women currently used iq’mik in this region.

**Conclusion:**

Our results suggest that different types of tobacco use are epidemic among diverse Alaska Native communities. Our results also illustrate that detailed analysis within racial/ethnic groups can be useful for public health programme planning to reduce health disparities.

The term Alaska Native is used to refer to the Indigenous inhabitants of the land that is now the state of Alaska. The more than 138,000 Alaska Native people (single or multi-race) now living in Alaska make up about 24% of its residents (1). The state of Alaska is one-fifth the size of the continental United States, and many areas of the state can only be reached by boat or plane (2). Alaska’s Native people have historically been geographically distant from one another and thus have grown culturally diverse. The more than 200 tribes fall into 5 distinct Alaska Native cultural subgroups: Inupiaq; Athabascan; Yup’ik and Cup’ik; Aleut and Alutiiq; and the Eyak, Tlingit, Haida and Tsimshian (3). However, despite this diversity, most reports published in the US provide health behavior estimates only for “American Indian/Alaska Native” people combined across the nation (4). In a Surgeon General’s report (5) that presented smoking prevalence among different American Indian/Alaska Native (AIAN) subgroups, Alaska Native people had the highest reported smoking prevalence among all AIAN or Native American cultural groups.

The *Nicotiana* tobacco plant is not a naturally occurring plant to Alaska, and therefore did not historically have traditional significance for Alaska Native people, as it does for some other Native American groups (6); however, commercial tobacco use is now common among Alaska Native people. In a recent report by Alaska’s Department of Health to describe tobacco use specifically among Alaska Native people, prevalence among Native peoples was significantly greater than, and approximately twice the prevalence among non-Native Alaskans (7). For example, in 2005, 40.6% of Native versus 22.0% of non-Native Alaskan adults smoked cigarettes, and 10.8% of Native versus 2.0% of non-Native Alaskan adults used smokeless tobacco (SLT). Kim et al. used a statewide survey of women who had recently delivered a child and reported that more than 40% of Alaska Native women used some form of tobacco during pregnancy (8). Angstman et al. found 12% current SLT use among adolescents aged 6–10 and 44% among aged 15–18 using medical records from the Yukon–Kuskokwim Native Health Corporation (9). These findings provide ample evidence that Alaska Native people deserve intensive support for tobacco control, and further analysis is needed to understand patterns of tobacco use within the population.

In addition to commercial tobacco products, a number of reports, beginning in the 19th century, have described the use of an SLT variant called “iq’mik” (pronounced “ick-mick”) or “Blackbull” that is unique to Alaska Native communities in the southwest region of the state (6,10–13). Iq’mik is prepared by burning a woody fungus (*Phellinus igniarius*) from birch trees, and mixing the ash with leaf tobacco. The ash is mixed with tobacco leaves, pre-chewed in the mouth or mixed with water, and stored in containers to use later. Iq’mik is frequently shared among families; parents in some regions reportedly introduce children to use early, including as a teething remedy for infants (6,11). This early introduction of iq’mik to young children may explain results from a study of 3–6-year-old preschoolers, where 3.5% were found to have saliva cotinine levels far exceeding levels consistent with secondary exposure, and suggestive of primary tobacco use by the children (14). Perham-Hester used a statewide survey of women who had recently delivered a child and reported that 4.1% of Alaska Native women statewide had used iq’mik during pregnancy (15).

Not surprisingly, rates of tobacco-related diseases such as lung and mouth cancers (16–18), heart disease and stroke (19), and chronic obstructive pulmonary disease (COPD) (20) are also greater among Alaska Native people than among non-Natives in Alaska or US Whites. Additionally, excess rates of infant death and illness among Alaska Native people can be partially attributed to prenatal smoking and second-hand smoke exposure (21). Some of these studies have called for more detailed descriptive studies of Alaska Native health risk behaviors by geographic area and ethnicity to aid in planning interventions (19).

The purpose of our study is to describe tobacco use rates for different types of tobacco among Alaska Native people, including in specific sub-regions of the state. Our study is the first to provide population-based statewide and regional estimates for tobacco use, including iq’mik, among the general population of Alaska Native adults.

## Methods

We used data from Alaska Native people included in the Alaska Behavioral Risk Factor Surveillance System (BRFSS) for 2006–2010 combined. BRFSS is an anonymous telephone survey of adults conducted by the Alaska Division of Public Health since 1991 in cooperation with the Centers for Disease Control and Prevention (CDC). The survey includes questions about health-related behaviors and health status. Interviews are conducted throughout the year.

The BRFSS uses a random digit dial method to select a representative sample of Alaska adults (aged 18 and older). The state sample is stratified into 5 regions, with roughly equal numbers of interviews conducted in each region. One survey respondent from each selected household is randomly chosen from among the adults living in the household. People without home-based telephones are not eligible for sampling (that is, persons living in dormitories, military housing, prisons, nursing homes and other institutional settings). Cell phones are not available for sampling, so individuals who use only cell phones as their home telephone are ineligible. Alaska’s BRFSS is administered only in English.

### Measures

#### Alaska Native race

We identified Alaska Native respondents as people who reported their race as “American Indian or Alaska Native” alone, or as their preferred race. Although the survey response option is phrased as “American Indian or Alaska Native” (AIAN), we use the term “Alaska Native” in this paper because most AIAN people living in Alaska more specifically identify as Alaska Native, and this is the language commonly used in Alaska, and by Native organizations, to refer to the Indigenous people living in the state (22).

#### Demographic characteristics

Respondents provided their exact age and highest level of formal education completed. They also provided information about total household income (estimated), and whether there were children in the home.

#### Geographic region

We created geographic regions based on service areas for the state’s Native Health Corporations (tribal and Native health organizations that provide health services and related programmes). Individuals were assigned to a region based on telephone prefix, which is linked to specific geographic areas in Alaska.

#### Cigarette smoking

Respondents who had smoked at least 100 cigarettes in their lifetime and currently smoke “every day” or “some days” were coded as current smokers.

#### Smokeless tobacco

Respondents who said that they currently used any SLT products such as chewing tobacco or snuff, iq’mik or Blackbull were classified as current users.

#### Iq’mik

We classified respondents as iq’mik users if they responded “yes, iq’mik or Blackbull” to the BRFSS question “Do you currently use any smokeless tobacco products such as chewing tobacco or snuff, iq’mik, or Blackbull?” Unfortunately, one of the response options to the question about SLT was “more than one” and since it would be possible to give this answer and not use iq’mik, we did not classify people who gave this response as users and thus the true prevalence of iq’mik use may be higher than we are reporting here.

### Analysis

We used unweighted data to describe the sample population, and weighted the data for tobacco use prevalence estimates to adjust for sampling design (based on region and telephone listing), and for the number of telephones and adults in each household. Weighted data were also post-stratified to the age and sex distribution of the Alaska population.

We used the Pearson Chi-square test of independence to determine whether different types of tobacco use were associated with distributions of age, gender, education, income and having children in the home. We stratified by Alaska Native Health Corporation regions, combining smaller regions so that there were at least 50 respondents in any group, to describe geographic patterns of tobacco use among Alaska Native people. Analyses were completed using Stata/IC 10.1^®^, and using a significance level of 0.05.

## Results

Table I describes the unweighted characteristics of the 4,143 Alaska Native adults included in Alaska’s BRFSS in the years 2006–2010. About half of the respondents were under age 45, and more than half had children living in the home. Approximately one-third had any college education and about 3 out of 4 reported a household income of less than $50,000 per year. Respondents were spread throughout 12 regions of Alaska.

**Table I:** Characteristics of Alaska Native adults, Alaska BRFSS 2006–2010 (N=4,143)

Characteristics	N	Percent
Age		
18–24	409	10.1
25–34	784	19.4
35–44	816	20.1
45–54	938	23.2
55–64	682	16.8
65 and above	423	10.4
Gender		
Male	1,842	44.5
Female	2,301	55.5
Highest education		
Less than high-school graduate	815	19.8
High-school graduate or GED	1,940	47.1
College 1–3 years	977	23.7
College graduate	390	9.5
Household income		
Less than $15,000	741	22.1
$15,000–24,999	762	22.8
$25,000–49,999	909	27.2
$50,000–74,999	428	12.8
$75,000 or more	508	15.2
Children in the home		
No children in home	1,307	41.0
Children living in the home	1,883	59.0
Alaska Native Health Corporation‡		
Aleutians and Pribilofs	104	2.5
Anchorage/Mat-Su	307	7.4
Arctic Slope	176	4.3
Bristol Bay	302	7.3
Copper River/Prince William Sound	70	1.7
Interior	553	13.4
Kenai Peninsula	213	5.1
Kodiak	121	2.9
Northwest Arctic	315	7.6
Norton Sound	373	9.0
Southeast	635	15.3
Yukon–Kuskokwim	973	23.5

‡ Assigned by telephone prefix.

Table II shows tobacco use prevalence among different subgroups. Approximately 2 out of every 5 Alaska Native adults reported smoking cigarettes (41.2%). About 1 in 10 reported using some type of SLT (12.3%), and a relatively small percentage statewide (4.8%) reported using iq’mik alone. When cigarettes and SLT were combined, about half of Alaska Native adults were currently using some type of tobacco product.

**Table II:** Tobacco use among Alaska Native adults by demographic group, Alaska BRFSS 2006–2010

	Cigarettes			Any smokeless			Iqmik alone			Any tobacco		
	
	%	(95% CI)	p	%	(95% CI)	p	%	(95% CI)	p	%	(95% CI)	p
All Alaska Native adults	41.2	(38.7–43.7)		12.3	(11.0–13.7)		4.8	(4.1–5.6)		51.3	(48.8–53.8)	
Age												
18–24	47.4	(40.0–55.0)		12.0	(8.7–16.2)		4.8	(2.9–7.7)		55.9	(48.1–63.4)	
25–34	51.8	(45.7–57.8)		14.4	(11.1–18.3)		4.3	(3.0–6.2)		61.7	(55.8–67.3)	
35–44	40.2	(35.6–45.0)		15.7	(12.9–18.9)		6.2	(4.6–8.3)		52.9	(47.8–57.9)	
45–54	38.8	(34.5–43.2)		12.2	(9.7–15.1)		5.2	(3.7–7.2)		50.5	(45.8–55.2)	
55–64	34.0	(29.2–39.2)		10.2	(7.3–14.1)		5.0	(3.2–7.6)		43.0	(37.9–48.3)	
65 and older	21.1	(15.9–27.5)	<0.001	4.3	(2.6–7.1)	0.002	1.8	(0.8–3.8)	0.17	27.1	(21.2–33.8)	<0.001
Gender												
Male	44.7	(40.9–48.5)		15.7	(13.6–18.2)		4.1	(3.2–5.3)		56.0	(52.2–59.8)	
Female	37.6	(34.6–40.6)	0.004	8.7	(7.4–10.3)	<0.001	5.5	(4.4–6.8)	0.08	46.4	(43.2–49.6)	<0.001
Highest formal education												
Less than high-school graduate	48.7	(43.1–54.3)		15.3	(12.1–19.1)		6.7	(4.9–9.2)		59.7	(54.0–65.2)	
High-school graduate or GED	45.4	(41.7–49.2)		14.7	(12.7–17.0)		6.0	(4.8–7.4)		57.3	(53.6–60.9)	
College 1–3 years	35.4	(31.0–40.1)		8.3	(6.2–11.2)		2.4	(1.6–3.8)		43.7	(39.0–48.6)	
College graduate	20.8	(15.2–27.8)	<0.001	5.3	(3.1–9.0)	<0.001	1.3	(0.4–3.8)	<0.001	26.2	(20.0–33.5)	<0.001
Household income												
Less than $15,000	48.5	(42.5–54.7)		17.2	(13.7–21.3)		8.4	(6.2–11.2)		60.8	(54.5–66.7)	
$15,000–24,999	49.3	(43.8–54.9)		12.6	(9.7–16.1)		5.0	(3.6–7.1)		57.8	(52.2–63.2)	
$25,000–49,999	40.9	(35.5–46.5)		11.9	(9.2–15.2)		5.1	(3.5–7.3)		51.1	(45.5–56.6)	
$50,000–74,999	36.9	(29.2–45.3)		8.2	(5.6–11.9)		1.4	(.5–3.7)		45.3	(37.3–53.6)	
$75,000 or more	27.4	(20.4–35.7)	<0.001	4.0	(2.6–6.1)	<0.001	0.1	(0–.7)	<0.001	31.3	(24.0–39.6)	<0.001
Children in the home												
No children in home	36.1	(32.2–40.1)		7.7	(6.0–9.8)		2.3	(1.5–3.7)		43.4	(39.2–47.7)	
Children living in the home	45.2	(41.3–49.1)	0.001	15.4	(13.3–17.8)	<0.001	6.8	(4.3–6.1)	<0.001	57.3	(53.5–61.1)	<0.001

Estimates weighted to adjust for sampling design, and post-stratified to state population for age and gender.

Both cigarette smoking and SLT use were significantly associated with age, gender, education, income and having children in the home. Highest smoking prevalence was measured among younger adults (51.8% among people aged 25–34), men (44.7%), people with the least years of formal education (48.7% among those with less than high-school education), lowest household income (48.5% among people in households with less than $15,000 per year), and people with children in the home (45.2%).

SLT use prevalence was highest among middle-aged people (15.7% among 35–44), men (15.7%), people with less formal education (15.3% among people with less than high-school education), lowest income (17.2% among people in households with less than $15,000 per year) and people with children in the home (15.4%).

Iq’mik use was not significantly associated with age or gender, but was significantly associated with education, income and having children in the home. Iq’mik use prevalence was highest among people with less formal education (6.7% among people with less than high-school education), lowest income (8.4% among people with household income less than $15,000) and people with children living in the home (6.8%).

Use of either cigarettes or SLT was significantly associated with all demographic characteristics, with more than half of Alaska Native adults using some form of tobacco in several subgroups: adults younger than 55 years, males, people with a high-school education or less, people with household income less than $50,000 per year and people who had children in the home.

**Fig. 1:**
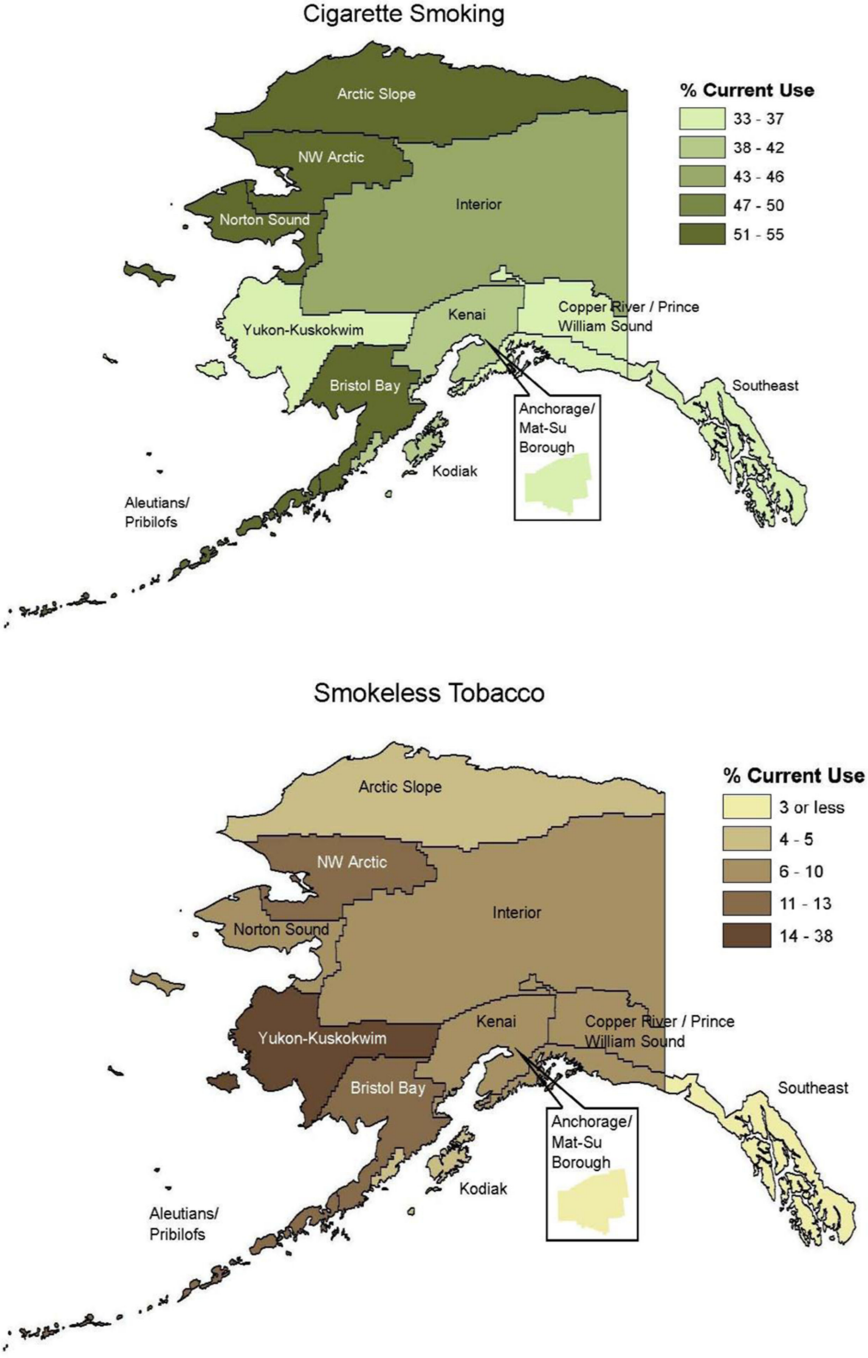
Prevalence of current tobacco use among Alaska Native adults, by Alaska Native Health Corporation Region.

Table III shows the prevalence of cigarettes, any SLT, iq’mik alone, and all tobacco combined, stratified by geographic region. Figure 1 shows maps for ranges of cigarette and SLT use across Alaska’s regions.

**Table III:** Tobacco use among Alaska Native adults by Alaska Native Health Corporation Region, Alaska BRFSS 2006–2010

	Cigarettes		Any smokeless		Iqmik alone		All tobacco combined	
	
	Percent*	(95% CI)	Percent*	(95% CI)	Percent*	(95% CI)	Percent*	(95% CI)
Alaska Native Health Corporation‡								
Aleutians and Pribilofs	53.6	(42.7–64.2)	13.0	(6.8–23.3)	1.7	(0.4–7.6)	61.3	(50.1–71.4)
Anchorage/Mat-Su	37.3	(30.1–45.0)	2.7	(1.3–5.6)	0.1	(0–0.4)	39.5	(32.3–47.2)
Arctic Slope	54.6	(45.4–63.4)	4.5	(1.9–10.4)	0		60.7	(51.3–69.4)
Bristol Bay	50.3	(42.7–57.9)	13.0	(8.7–19.0)	1.6	(0.6–4.1)	57.1	(49.0–64.7)
Copper River/Prince William Sound	32.8	(21.1–46.9)	9.1	(3.3–22.6)	1.9	(0.3–12.5)	42.3	(28.7–57.1)
Interior	42.7	(37.6–48.0)	9.5	(6.8–13.0)	0.1	(0–0.4)	51.0	(45.8–56.2)
Kenai Peninsula	38.4	(30.6–46.7)	9.7	(5.5–16.3)	2.5	(0.6–9.5)	47.2	(38.9–55.7)
Kodiak	38.1	(27.8–49.6)	5.3	(2.3–12.2)	0.6	(0.1–4.3)	44.8	(33.9–56.3)
Northwest Arctic	52.5	(45.7–59.2)	12.1	(7.7–18.4)	0.5	(0.1–3.4)	62.0	(55.2–68.3)
Norton Sound	52.3	(46.2–58.4)	10.4	(6.4–16.4)	0.8	(0.2–3.1)	63.5	(57.2–69.3)
Southeast	36.6	(32.2–41.3)	2.7	(1.4–5.3)	0		39.9	(35.3–44.7)
Yukon–Kuskokwim	36.4	(32.5–40.5)	37.5	(33.6–41.6)	23.3	(20.0–26.9)	66.4	(62.7–69.9)

* Estimates weighted to adjust for sampling design, gender and age.

‡ Assigned by phone prefix.

More than half of Alaska Native adults in the Aleutians/Pribilofs (53.6%), Arctic Slope (54.6%), Bristol Bay (50.3%), Northwest Arctic (52.5%) and Norton Sound (52.3%) health corporation regions reported current smoking. The lowest smoking prevalence, 32.8%, was reported in the Copper River/Prince William Sound region.

More than one-third of Alaska Native adults in the Yukon–Kuskokwim (Y–K) region reported current use of SLT (37.5%), which was significantly higher than for any other region of the state; Anchorage/Mat-Su (2.7%) and Southeast (2.7%) had lower SLT use prevalence than many other regions of the state. SLT use prevalence ranged from 4.5 to 13% in other regions of the state. Use of iq’mik was almost entirely confined to the Y–K region (23.3%).

When combining cigarette and SLT use, only 5 of the 12 regions had less than half of the adults reporting current use of tobacco. The highest prevalence was measured in the Y–K region, where about two-thirds (66.4%) of adults reported using some type of tobacco.

Finally, we explored data from the Y–K region alone to examine factors associated with iq’mik use, since iq’mik use was primarily confined to that region. Women in the Y–K region were significantly more likely than men to use iq’mik (30.1% vs. 17.8%; data not shown). Higher income was associated with decreased prevalence of iq’mik use (23.4–27.1% among people with less than $50,000 household income per year vs. 1.6–16.8% among people with more than $50,000 per year). Iq’mik use in the Y–K region was not significantly associated with age, having children in the home, or highest level of formal education, although small numbers may have prevented us from detecting associations.

## Discussion

In this study, we were able to identify different patterns of tobacco use by Alaska Native adults in different regions of the state, and among different demographic subgroups. We organized the information in this study according to geographic regions served by Alaska Native Health Corporations, so that the results would be as relevant and portable as possible for use by stakeholders prioritizing and planning effective programmes to support Alaska Native people.

Having detailed information to describe patterns of tobacco use is an important component of working to decrease use and disparities. For example, health goals were established specifically for Alaska Native people as part of the state’s “Healthy Alaskans 2010” initiative. These goals included reducing smoking to 14% or less, and reducing the use of SLT to 3% or less (from respective baselines of 42 and 12% among Alaska Native adults statewide in 1999) (23). It seems unlikely that these goals have been met. Understanding what segments of the Alaska Native population are at greatest risk will help to achieve these ambitious and important goals.

The prevalence of cigarette smoking among Alaska Native people was high in all regions, but prevalence was highest in some of the most remote areas of Alaska. Smoking prevalence was lower in the relatively more urban Anchorage/Mat-Su and Southeast (Juneau) areas. These areas are also where proven tobacco control interventions such as tax increases and smoke-free workplaces have been most aggressively applied: although not designed to reach Alaska Native people specifically, such interventions may influence all people who live there. This difference in regional prevalence may be related both to the difficulty in sufficiently funding and supporting programmes across the vast state, and to translating “best practice” tobacco control interventions (such as policies and healthcare interventions) to frontier village environments. However, because so many Alaska Native people live in such environments, it is highly unlikely that overall goals for reducing tobacco use and improving population health can be met unless these programmes are adapted or re-conceptualized to be effective in very rural settings.

The Y–K region shows a particularly unique pattern of tobacco use: although among the lower prevalence regions for cigarette smoking, it was among the highest region for SLT use. Furthermore, as had been reported in a small number of isolated studies, we confirmed empirically that the use of iq’mik was primarily concentrated in the Y–K region. Upon further stratification, we found that approximately 1 in 3 Alaska Native women in the Y–K region reported using iq’mik, significantly more than men. This is consistent with other reports of high prevalence of iq’mik use among women of childbearing age in Southwest Alaska (12,13,24). Our findings provide additional evidence from a population-based public health surveillance system of the need for support to reduce tobacco use, especially SLT use, among Alaska Native women in this area of the state.

Wolsko et al. (25) found that the use of iq’mik was more highly prevalent among Yup’ik adults practicing traditional lifestyles, while cigarette smoking was more highly prevalent among Yup’ik adults practicing Western lifestyles. We did not have measures of cultural lifestyle in our study, but we noted that the use of iq’mik was not significantly associated with age, in contrast to smoking (which showed a more typical pattern of higher use among young adults). This pattern could be explained by differences in lifestyle practices, if younger adults are less likely to practice traditional lifestyles than older adults. Future development of some measures of traditional versus Western lifestyle practice may be useful for better understanding the association between health-related practices and diverse cultures.

Although we described tobacco use separately for cigarettes and SLT, it is also important to understand individual practices for blending or switching behaviors. Focus group participants in other studies have reported that men often switch from iq’mik to cigarettes as adults, while women continue using iq’mik, and yet others may switch to iq’mik when quitting cigarettes or if cigarettes are not available (11). Patten (26) recently documented a surprising increase in the prevalence of (any) SLT use from 14% pre-pregnancy to 60% during pregnancy in the Y–K health corporation population. We found that the use of multiple tobacco products was frequent among Alaska Native adults in some areas of the state. Also, we noted that SLT use was higher among people with children in the home. Interventions may benefit from anticipating tobacco type-switching behaviors, and exploring the reasons behind them, in Alaska Native communities.

We found different patterns of tobacco use among Alaska Native adults in different regions of the state, but we did not find any areas of the state where tobacco interventions were not needed. Huge gaps exist in the identification of culturally appropriate and effective health promotion programmes, and ways to disseminate those programmes (27). Past efforts to reduce tobacco use specifically among Alaska Native people have included the integration of tobacco cessation clinical best practices into Alaska Native health corporation systems that provide health services to Alaska Native people (28), targeted culturally appropriate education programmes such as the *Traditions of the Heart* cardiovascular disease screening and education programme for under-insured Alaska Native women (29) and funding community-based programmes in rural areas largely populated by Alaska Native people. To address the problem of tobacco, some communities have implemented highly successful campaigns to implement local tobacco taxes, which are effective for preventing youth from starting to use tobacco and for helping adults to quit (30). Notably, the largely Alaska Native community of Bethel implemented a $2.21 per-pack tobacco tax in February 2013, placing Bethel among the top 10 in the nation for the application of price interventions; other Alaska communities including Anchorage, Barrow, Matanuska-Susitna Borough, Sitka, Juneau, and Fairbanks also rank among the leading communities in the nation (31). Future data collection and analyses may reveal the anticipated benefits of these recent interventions.

### Limitations

Data from the Alaska BRFSS used in this report may not accurately represent the whole Alaska Native population. For example, Schumacher et al. (32) reported that 8% of Alaska Native respondents in the EARTH study spoke only Alaska Native languages. These Alaska Native people would not be included in BRFSS, which is available only in English, and their tobacco use patterns may be different than those of people who speak English (alone or in combination with other languages). The survey also excludes people without a telephone landline, who may have different patterns of tobacco use.

Tobacco use may be underestimated in our study because people might be reluctant to report behaviors/attitudes that others might not find acceptable (particularly over the phone to a stranger). Information from community stakeholders suggests that Alaska Native people may in particular be uncomfortable participating in telephone surveys and revealing personal information over the telephone.

Our measure of iq’mik use was problematic because one response option to the question “what type of smokeless tobacco do you use?” was “more than one product.” It is very likely that some people use iq’mik in combination with commercial SLT or snuff, and these people would not have been classified as iq’mik users in our analysis because we could not confirm their use. If all of the “multiple product” users were also using iq’mik, the real prevalence of iq’mik use may be higher than reported here.

## Conclusions

Tobacco use is highly prevalent among Alaska Native people, throughout Alaska, but patterns of product use are different in different regions. These unique patterns of tobacco use may be considered when deploying interventions to reduce tobacco use regionally or statewide, and suggest that more culturally appropriate interventions are needed to address specific products, and for rural communities. Continued monitoring of trends in tobacco use for different regions of the state may help to identify areas of the state that are successful in reducing tobacco use and inform the evolution of “best practices” for Alaska Native communities. Our findings also illustrate the importance and utility of conducting descriptive investigations within subpopulations of a racial/ethnic minority subgroup.
